# Semantic knowledge influences visual working memory in adults and children

**DOI:** 10.1371/journal.pone.0241110

**Published:** 2020-11-11

**Authors:** Ariel Starr, Mahesh Srinivasan, Silvia A. Bunge

**Affiliations:** 1 Department of Psychology, University of Washington, Seattle, Washington, United States of America; 2 Department of Psychology, University of California, Berkeley, Berkeley, California, United States of America; Zhejiang Univeristy, CHINA

## Abstract

We can retain only a portion of the visual information that we encounter within our visual working memory. Which factors influence how much information we can remember? Recent studies have demonstrated that the capacity of visual working memory is influenced by the type of information to be remembered and is greater for real-world objects than for abstract stimuli. One explanation for this effect is that the semantic knowledge associated with real-world objects makes them easier to maintain in working memory. Previous studies have indirectly tested this proposal and led to inconsistent conclusions. Here, we directly tested whether semantic knowledge confers a benefit for visual working memory by using familiar and unfamiliar real-world objects. We found a mnemonic benefit for familiar objects in adults and children between the ages of 4 and 9 years. Control conditions ruled out alternative explanations, namely the possibility that the familiar objects could be more easily labeled or that there were differences in low-level visual features between the two types of objects. Together, these findings demonstrate that semantic knowledge influences visual working memory, which suggests that the capacity of visual working memory is not fixed but instead fluctuates depending on what has to be remembered.

## Introduction

Visual working memory is a system that enables us to maintain and manipulate visual information in our minds [1]. One of the hallmark features of visual working memory is that it is capacity-limited: it does not consist of the entire contents of the visual information we encounter, but instead only a subset [2]. Classic studies of visual working memory using simple shapes or colored squares have demonstrated that the average person can maintain approximately four items in working memory [3].

However, recent research has demonstrated that visual working memory capacity is influenced by the type of visual information to be remembered [4–9]. For example, Brady and colleagues [5] had adult participants perform a change detection task with either colored squares or pictures of real-world objects, with an encoding window that varied between 200 and 2000 ms. With 200 ms of encoding time, visual working memory capacity was similar for colored squares and real-world objects. As encoding time increased, however, memory performance for colored squares remained flat, whereas memory for real-world objects continued to improve. This benefit for real-world objects when given increased encoding time was hypothesized to stem from the retrieval of semantic long-term memory representations associated with these objects, which are believed to reinforce and strengthen the representations of these objects in working memory [5]. If this hypothesis is correct, the mnemonic benefit for objects should be stronger for objects with which participants are familiar, because some level of familiarity with an object is needed in order to possess semantic knowledge about it. The present studies address this hypothesis and provide insight into the influence of semantic knowledge on visual working memory in both adults and children.

Previous work has provided mixed support for the proposal that semantic knowledge or item familiarity influence working memory. On the one hand, studies that have induced familiarity through repeated exposure to novel polygons or geometric patterns have found no benefit in working memory for the familiar versus novel stimuli [10, 11]. On the other hand, a number of studies have found that expertise with a particular category leads to enhanced visual working memory performance for visual stimuli from that category [6, 9, 12, 13]. For example, Jackson and Raymond [12] found that visual working memory performance was significantly better for famous versus unfamiliar faces and concluded that visual working memory is facilitated when visual representations of the to-be-remembered items are present in long-term memory. One explanation for these seemingly conflicting results is that perceptual familiarity in the absence of any context or semantic richness (e.g., as in the case of novel polygons) does not lead to the type of robust representations in long-term memory that can support visual working memory. At the same time, there may also be limits on the generalizability of findings from studies involving domain experts, because these studies have examined the effect of familiarity on subordinate-level memory, with participants needing to discriminate between highly similar items within a specific category (e.g., famous faces or cars). It is thus unclear whether these results would extend to situations in which the to-be-remembered items represent familiar objects from a diverse range of categories. In addition, based on these prior studies it is unknown how much experience or familiarity one must have with an object for it to have an advantage in visual working memory.

Interestingly, a number of prior studies have also demonstrated that semantic or conceptual knowledge enhances memory in infants. The majority of these studies use the individuation and identity tracking task [14], which tests infants on their ability to determine whether one or two objects are hidden behind an occluder. In this paradigm, older infants correctly recognize that there are multiple objects present when the objects belong to different categories, and younger infants succeed when these contrasting categories are highlighted within the experiment [14–17]. Together, these results demonstrate that semantic knowledge helps infants individuate objects within an array, which is an important precursor for success on working memory change detection tasks [18]. However, these findings do not tell us whether having semantic knowledge about to-be-remembered items might actually strengthen the representations of these items in working memory after they have been individuated.

Another phenomenon consistent with the hypothesis that semantic knowledge influences visual working memory capacity, broadly construed, is chunking [19]. Chunking is a method that increases working memory capacity by re-encoding multiple items into a single unit, hence minimizing the total number of items that need to be remembered. Chunking has a robust effect on working memory capacity in adults (see [2] for review), and even infants are able to use their knowledge of familiar categories to improve their visual working memory capacity by chunking arrays of toys [20]. However, although these prior studies suggest that children and adults can use semantic knowledge to perceptually or conceptually group items, they leave open the question of whether semantic knowledge can strengthen the working memory representations of *individual items*, leading to higher working memory capacity.

In summary, though previous work provides indirect evidence that semantic knowledge may facilitate visual working memory, this proposal has not been directly tested. Here, we explicitly test this hypothesis by comparing visual working memory performance for arrays of familiar versus unfamiliar objects in a standard change detection task [3] in both adults and children to assess how varying levels of experience with familiar objects might influence visual working memory. In Experiment 1, adult participants performed a change detection task with pictures of either familiar or unfamiliar objects. To control for the possible effect of verbal labeling strategies, participants performed a concurrent verbal task that taxes phonological working memory. To control for possible differences in visual features between the familiar and unfamiliar images, participants performed the task with regular images and with morphed versions of the images. The morphed images obscured their semantic identity while preserving their visual features. Next, in Experiment 2 we tested whether children would also show a mnemonic benefit for familiar compared to unfamiliar objects. We tested two groups of children, one older (aged 6–9 years) and one younger (aged 4–5 years), to determine if this effect would be influenced by amount of exposure to the familiar objects. Together, our findings demonstrate that object familiarity influences visual working memory capacity in children and adults alike.

## Experiment 1

### Method

#### Participants

Nineteen participants completed Experiment 1 (mean age = 20.7 years, range = 18.4–23.9 years, 15 female). A target sample size of 18 subjects was chosen based on power analyses of the effect size found in Brady et al. (2016), and recruitment ended after at least this number of participants had signed up for the experiment. The Cohen’s *d* for the difference in memory capacity between objects and colors with a 2000 ms encoding window in Brady et al. [5] was approximately 0.9. Assuming a slightly smaller effect size of .8, 18 participants give us nearly 90% power to detect the effect. Participants gave written informed consent to Protocol #2013–08–5546 “Language and Cognition in Children and Adults,” which was approved by the University of California, Berkeley Committee for Protection of Human Subjects and were compensated with partial course credit.

#### Procedure

Participants performed a change detection task that manipulated image familiarity and morph status in a within-subjects design. Participants completed four blocks of 40 trials each. Within each block, image familiarity and morph status were held constant. Two blocks contained regular images, and two blocks contained morphed images. Blocks were presented in a pseudorandom order, such that image familiarity alternated between blocks.

**Fig 1:**
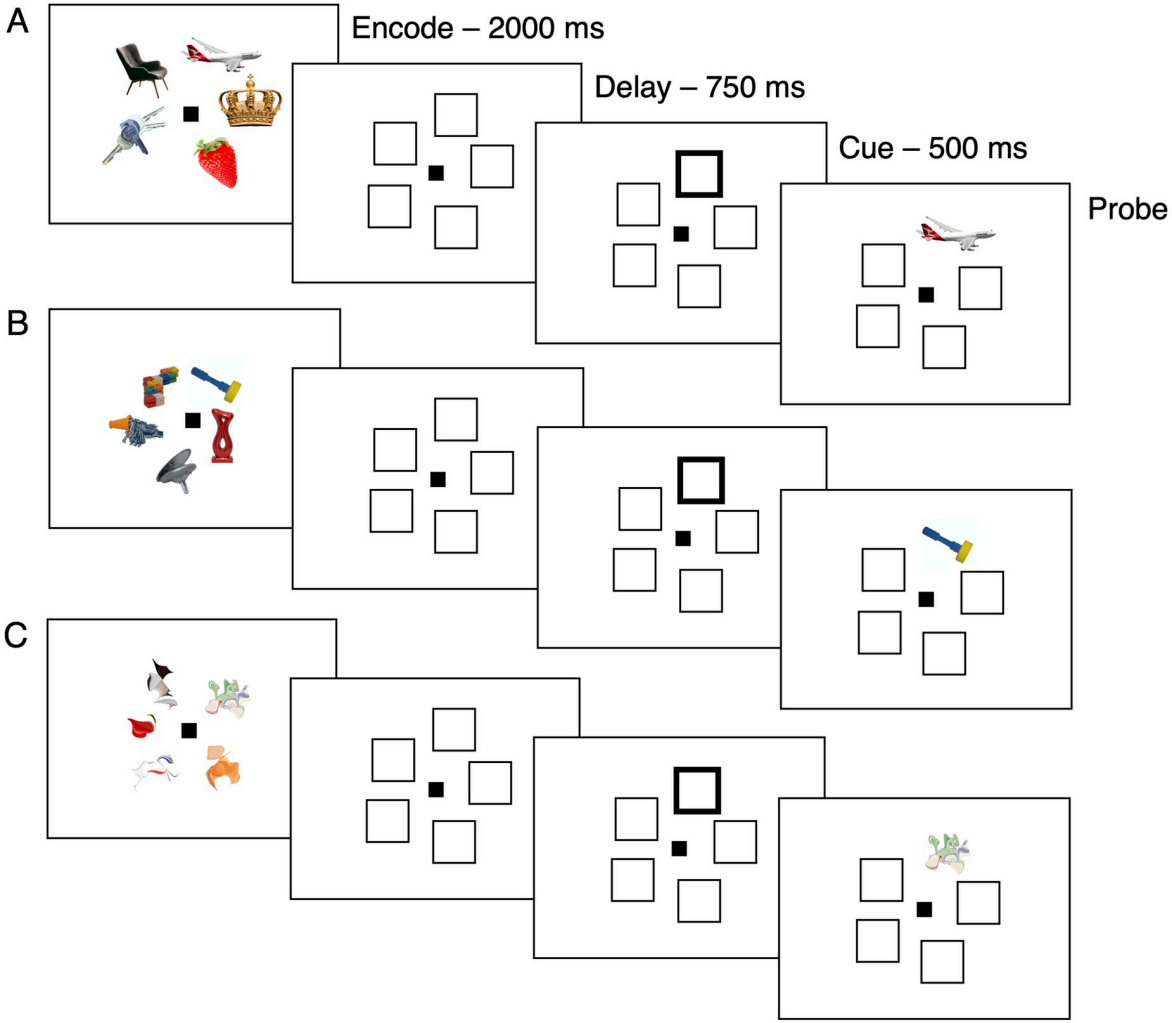
Schematic of the change-detection task. (A) Example familiar images. (B) Unfamiliar images. (C) Morphed images. Images in (A) are similar to but not identical to those used in the experimenter and are for illustrative purposes only. Images in (B) are reprinted under a CC BY license with permission from Horst (2016).

Participants saw an array of five items (familiar or unfamiliar pictures) that appeared on the screen for 2000 ms. After a 700 ms delay, one location was cued for 500 ms, after which the probe item appeared in that location. Participants were instructed to press ‘S’ if the item was the same as the one that had been shown in the cued location, and ‘D’ if the item was different (Fig 1). Within each block there were an equal number of same and different trials. Stimulus presentation and response collection were controlled by Matlab on a 15” Mac laptop.

Participants also performed a concurrent phonological working memory task that involved repeating two digits aloud while performing the change detection task. Before each trial, the digits appeared on the screen and participants were requested to repeat the digits aloud for the duration of the trial. After the change detection trial, participants were instructed to type in the digits on the laptop. A new pair of digits would then appear before the next trial. An experimenter remained in the room with the participants to ensure that they repeated the digits aloud throughout the entire experiment.

#### Stimuli

All stimuli were color images of three-dimensional objects. The images where chosen from the stimuli pool from Brady et al. [5], the Novel Object and Unusual Name (NOUN) database [21], and Google image search. Familiar objects were pictures of common objects that should be familiar and nameable for children aged 4–6 years, based on picture naming norms from Robertson et al. [22] and language acquisition norms from Wordbank [23]. Unfamiliar objects were pictures of obscure objects and images from the NOUN database. Note that familiarity is used in the present study to indicate that participants are expected to have previously encountered the pictured object in the real world and be able to name it, not that they have seen the object previously within the experiment. Here, familiar items are items for which participants have semantic knowledge based on their lived experience, and unfamiliar items are obscure objects that participants are unlikely to have encountered in the world.

The relative familiarity of each pictured item was validated using a sample of 112 Amazon Mechanical Turk workers who answered the question “Have you seen this type of object before?” using a scale ranging from 1 (“Definitely not”) to 5 (“Definitely yes”). The mean familiarity score was 4.63 (95% CI = [4.59, 4.70]) for the familiar images and 2.51 [2.47, 2.56] for the unfamiliar images. As expected, the ratings for objects selected to be familiar and unfamiliar were significantly different from one another (two-sample t-test, *t*(2587.1) = 57.95, *p* < .001, Cohen’s *d* = 1.57). All images were trial-unique to minimize interference.

To determine if differences in low-level visual features might contribute to a familiarity effect, we had participants perform the change detection task with the standard sets of images and with images that had been diffeomorphed [24]–a method of scrambling images that preserves their basic visual properties while obscuring their semantic identity. The diffeomorphic transformation involves repeatedly applying a flow field generated from a set of two-dimensional cosine components with random phase and amplitude. The resulting images produce very similar simulated activation patterns to intact images using the HMAX standard model of object perception [25] but are semantically unrecognizable by humans [24].

#### Data analysis and management

Accuracy data from the change detection tasks were analyzed with mixed effects models using the lme4 and lmerTest packages in R [26, 27]. Follow-up comparisons were conducted using paired t-tests. All stimuli, raw data, and the RMarkdown script used to produce the results sections for both experiments can be found at https://osf.io/wmzpx/. In addition, a summary of four preliminary experiments that motivated Experiment 1 can be found in the S1 Materials.

### Results

**Fig 2:**
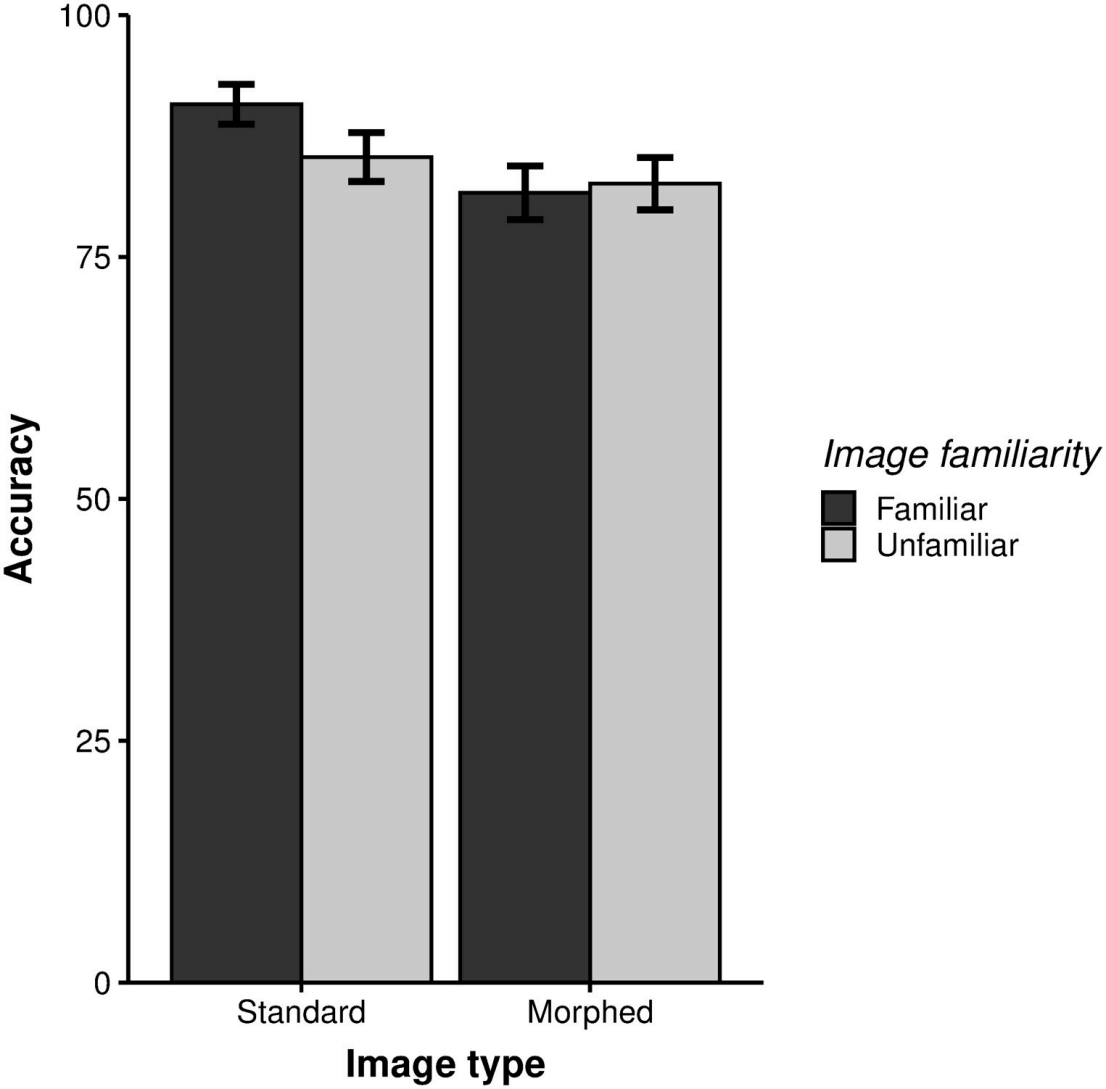
Visual working memory accuracy (percent correct). Adult memory performance for familiar versus unfamiliar objects in the standard and morphed image conditions. Error bars indicate SEM.

We analyzed memory accuracy data for each trial using a generalized logistic mixed effects model with image familiarity (familiar or unfamiliar), image type (standard or morphed), and their interaction as fixed effects, and subject as a random effect. This analysis revealed that the main effects and interaction were all significant (familiarity: *β* = -1.139, *p* = 0.001; image type: *β* = -0.812, *p* < .001; interaction: *β* = 0.604, *p* = 0.004; Fig 2). For standard images, memory accuracy was significantly better for familiar images compared to unfamiliar images (familiar: *M* = 90.78%, 95% CI [88.71–92.84%]; unfamiliar: *M* = 85.32% [82.79–87.85%]; paired t-test: *t*(18) = 3.28, *p* = 0.004, Cohen’s *d* = .763). For morphed images, however, memory accuracy did not differ between familiar and unfamiliar images (familiar: *M* = 81.62% [78.85–84.4%]; unfamiliar: *M* = 82.56% [79.85–85.27%]; paired t-test: *t*(18) = -0.61, *p* = .548, Cohen’s *d* = -.121). Similarly, visual working memory capacity, as measured by K [2], was significantly higher for familiar compared to unfamiliar images in the standard image condition (familiar: *K* = 4.08; unfamiliar: *K* = 3.53; paired t-test: *t*(18) = 3.28, *p* = .004, Cohen’s *d* = .723), but did not differ for between the two types of morphed images familiar: *K* = 3.16; unfamiliar: *K* = 3.26; paired t-test: *t*(18) = -0.65, *p* = .523, Cohen’s *d* = -.126). Digit recall accuracy was very high (*M* = 96.13%), which confirms that the participants were actively keeping the digits in mind while performing the change detection task. Taken together, these results demonstrate that there is a benefit in visual working memory for familiar compared to unfamiliar objects, and that this benefit does not stem from labeling strategies or differences in low-level visual features.

## Experiment 2

Experiment 1 demonstrates a mnemonic benefit in visual working memory for familiar compared to unfamiliar objects in adults that cannot be easily explained by differences in visual features of the stimuli or in the use of verbal labeling strategies. In Experiment 2, we investigated whether object familiarity would also benefit visual working memory in children. All of the objects in the familiar condition of Experiment 1 were chosen because they should be familiar to four-year-old children. However, children gain increasing experience with these objects over the years. Therefore, we tested both younger (aged 4–5 years) and older (aged 6–9 years) children with the standard images to investigate whether the familiarity advantage documented in adults might only emerge in our older group of children, after children have acquired many years of experience with the objects. So as to keep the overall length of the experiment suitable for children’s shorter attention spans, and because the familiarity effect was present only for the standard images in Experiment 1, we did not include blocks of morphed images in Experiment 2. In addition, because children in this age range are unlikely to spontaneously use verbal rehearsal strategies [28], children did not perform the concurrent verbal digit rehearsal task.

### Method

#### Participants

Data from 19 children aged 6–9 years (mean age = 8.2 years, range = 6.2–9.9, 12 female) and 25 children aged 4–5 years (mean age = 5.1 years, range = 4.3–5.9, 16 female) were included in the analyses. Data from an additional 6 children aged 4–5 years were excluded because these children either did not complete the task (n = 3) or provided the same response for every trial (n = 3). We aimed to match the sample size from Experiment 1, but a slightly larger sample was acquired in the younger age group so as to allow all eligible children within a preschool classroom to participate. Children were tested individually in a small room or in a quiet corner of their preschool. Parents or guardians of child participants gave written informed consent to Protocol #2013–08–5546 “Language and Cognition in Children and Adults,” which was approved by the University of California, Berkeley Committee for Protection of Human Subjects.

#### Procedure

Children were tested with a similar change detection paradigm to that used in adults in Experiment 1, but with reduced cognitive demands. For older children (aged 6–9), each array contained four items and the encoding duration was 2000 ms. Pilot testing suggested that this version of the task was too difficult for younger children. Therefore, for younger children (aged 4–5 years), each array contained 3 items and the encoding duration was 3000 ms. The timing of the delay, cue, and probe phases was the same as in Experiment 1; however, children did not perform the concurrent phonological working memory task. Older children responded by button press, and younger children provided a verbal response. Older children completed 30 trials in each block of familiar and unfamiliar objects, and younger children completed 15 trials per block. The order of the blocks was randomized across subjects.

#### Stimuli

The stimuli consisted of the standard images used in Experiment 1.

### Results

**Fig 3:**
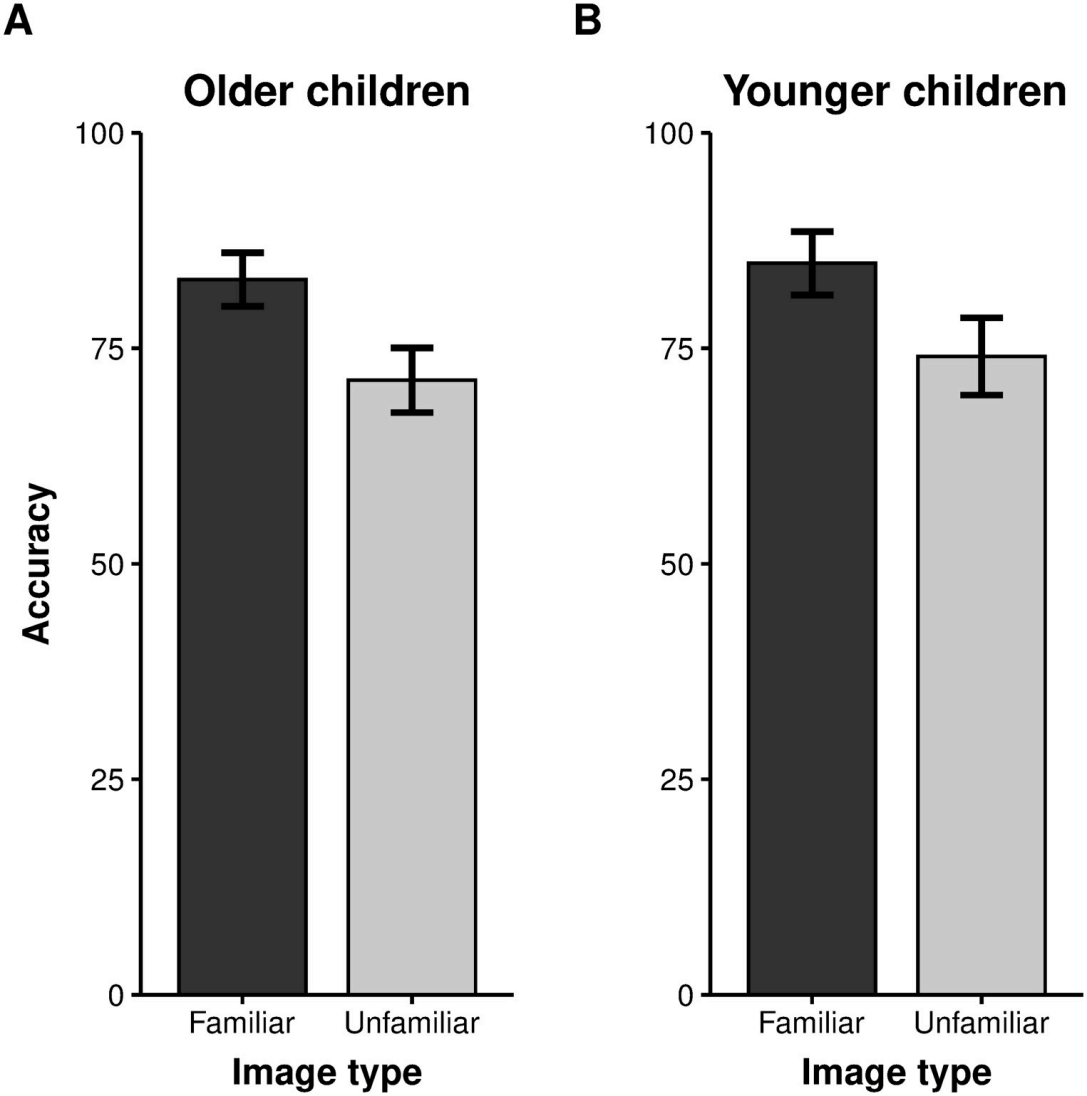
Memory accuracy by stimulus type (percent correct). (A) Older children (aged 6–9 years). (B) Younger children (aged 4–5 years). For older children, the memory arrays contained 4 items with an encoding duration of 2000 ms; for younger children, they contained 3 items with an encoding duration of 3000 ms. Error bars indicate SEM.

We analyzed accuracy data using paired t-tests to compare memory accuracy for familiar versus unfamiliar pictures in older and younger children separately (Fig 3). Older children exhibited better memory for familiar compared to unfamiliar pictures (familiar *M* = 82.98% [79.87–86.09%]; unfamiliar *M* = 71.3% [67.55–75.06%]; *t*(18) = 4.49, *p* < .001; Cohen’s *d* = 1.01), as did younger children (familiar *M* = 84.86% [81.2–88.53]; unfamiliar *M* = 74.05% [69.57–78.54%]; *t*(24) = 3.4, *p* = 0.002; Cohen’s *d* = 0.73). Visual working memory capacity was also significantly higher for familiar compared to unfamiliar pictures in both older children (familiar: *K* = 2.64; unfamiliar: *K* = 1.7, *t*(18) = 4.49, *p* < .001; Cohen’s *d* = 1.01) and younger children (familiar: *K* = 2.09; unfamiliar: *K* = 1.44, *t*(24) = 3.53, *p* = .002; Cohen’s *d* = .73). Together, these results demonstrate that children, like adults, have an advantage for maintaining familiar compared to unfamiliar objects in visual working memory.

## General discussion

The present studies demonstrate that visual working memory performance is influenced by semantic knowledge. Both children and adults are better able to remember familiar objects compared to unfamiliar ones, and this effect is already present in preschool-aged children. These results complement previous studies demonstrating that working memory capacity is influenced by the type of visual information to be maintained [4, 5, 7]. In addition, these findings extend prior work showing that experts in specific domains have enhanced visual working memory for items within that domain [6, 9, 12, 13, 29, 30] by showing that such expertise effects apply to representations of everyday objects with which most adults have years of experience. The present study also shows that by the time children begin preschool, they have already amassed enough familiarity with a range of objects to demonstrate this benefit.

Previous work has found a mnemonic benefit in working memory for real-world objects compared to colored squares, and has suggested that this benefit stems from semantic rather than episodic long-term memory systems [5]. In addition to calculating visual working memory capacity, Brady and colleagues [5] measured contralateral-delay activity, an electrophysiological marker of working memory maintenance that reflects the amount of information being actively held [31]. Critically, contralateral-delay activity drops when representations are consolidated into episodic memory and are no longer maintained in working memory [32]. Brady and colleagues [5] found that the greater visual working memory capacity for real-world objects compared to colored squares was also reflected in greater contralateral-delay activity. Because contralateral-delay activity scales with the amount of information being actively stored in visual working memory rather than reflecting processes in episodic memory [33], this finding suggests that the familiarity benefit for real-world objects stems from participants’ ability to draw on non-episodic long-term memory and perceptual representations in order to enhance visual working memory processes [5].

The present findings corroborate this hypothesis and demonstrate that participants must have semantic or perceptual representations of particular real-world objects in order for them to benefit from these enriched visual working memory processes. Additional support for an influence of semantic knowledge on working memory comes from a recent study that used ambiguous images to manipulate semantic content [34]. Participants exhibited greater working memory capacity and contralateral-delay activity for images that were perceived to be meaningful compared to images that were perceived to be meaningless. Although we did not measure contralateral-delay activity, we predict that this signal would be stronger on trials involving familiar than unfamiliar objects. This would suggest that semantic memory representations of familiar objects are activated during the process of working memory maintenance, and that these activated semantic representations strengthen the representations in working memory, resulting in the observed mnemonic benefit for familiar objects.

An alternative explanation for why having semantic knowledge about specific objects would improve visual working memory performance relates to the speed with which items can be encoded into working memory. A recent series of studies used participants’ experience with Pokémon characters to probe the effects of category expertise on visual working memory [9, 13]. These studies found that when the consolidation window was interrupted by the onset of a mask shortly after stimulus presentation, Pokémon experts were able to remember a larger number of familiar than unfamiliar Pokémon characters. Xie and Zhang interpreted this finding as indicating that familiar characters were consolidated more quickly into visual working memory–and that, as a result, a larger number of familiar characters could be maintained in working memory when encoding was interrupted. Thus, the familiarity benefit documented in the present studies could similarly be explained by faster encoding of familiar compared to unfamiliar objects. Further work is needed to determine the specific mechanism by which semantic knowledge influences visual working memory capacity. For example, eye-tracking could be used to test whether familiarity affects encoding efficiency, as measured by the number or duration of fixations participants make on familiar versus unfamiliar objects in an array.

The present findings are broadly consistent with theories of working memory that propose a role for long-term memory in supplementing the capacity of working memory, including Cowan’s [35] “virtual short-term memory” and Ericsson and Kintsch’s [30] “long-term working memory.” According to these theories, information held in long-term memory that is relevant to the current context can be activated to support information held in working memory. These theories provide explanations, for example, of why chess experts have superior memory for chess piece locations compared to novices, how individuals can memorize and recite hundreds or thousands of digits of Pi, and how grouping items on a grocery list into categories facilitates list recall. In general, these theories suggest that associations are created between the items in working memory and information held in long-term memory (e.g., canonical chess board layouts, categories of food), allowing for the grouping or chunking of items in working memory. Consistent with this prior work, the present study provides additional evidence that working and long-term memory are not fully independent. It also goes a step further, showing that having knowledge of an individual object results in a stronger working memory representation of that object–even in a time-limited context, in which explicit encoding strategies cannot be implemented.

In the real world, we frequently encounter objects that are neither entirely novel nor highly familiar–i.e., strength of familiarity lies on a continuum. Therefore, we might expect the effect of familiarity on visual working memory to also present as a continuous effect, such that the strength of the familiarity benefit in visual working memory scales with the level of object familiarity. On the other hand, it is possible that there is some threshold level of familiarity that must be crossed in order for a particular object to benefit from being familiar, such that the benefits of increasing familiarity are minimal once this threshold is crossed. In the present study, we found a mnemonic benefit for familiar objects across all age groups. However, because the age groups performed different variants of the task in terms of encoding duration and number of items per array, it is not possible to directly compare performance across the age groups and assess how the magnitude of the familiarity benefit changes with age. Because the present data cannot speak directly to this question, it is an interesting area for future study. In addition, the memory foil items in this study always belonged to a different category than the target; as a result, it is difficult to determine whether familiarity specifically enhanced memory for the exact item being maintained, or rather a category-level representation of that item. Follow-up work could address this question by using different exemplars from the same category as the target as foil items.

A wealth of prior research indicates that visual working memory undergoes dramatic development throughout childhood [e.g., 36–38], even after accounting for age-related differences in strategy and efficiency in allocating attention [39–41]. However, if semantic knowledge about objects contributes to visual working memory capacity, then it follows that age-related increases in semantic knowledge may also contribute to improvements in visual working memory capacity over development. In adulthood, our interactions with the world consist predominantly of interactions with objects that are familiar. However, this experience is quite different for young children, who are constantly encountering and learning about new objects. Our results suggest that interpreting differences in visual working memory across development requires that the type of stimuli used in the task be taken into account. More broadly, our findings suggest that the association between basic cognitive processes like working memory and later learning outcomes may in fact be bidirectional, such that greater working memory facilitates knowledge acquisition and greater semantic knowledge also enhances working memory.

In conclusion, we found evidence for a mnemonic benefit for familiar compared to unfamiliar objects, which demonstrates that semantic knowledge boosts visual working memory performance. This benefit was observed even in preschool-aged children, which supports the idea that increases in semantic knowledge could contribute to growth in working memory capacity over development. The idea that prior knowledge can exert an influence on working memory by providing a way to hierarchically organize the contents of memory has long been noted [2, 19]. However, the present results suggest that semantic knowledge can influence working memory not only by allowing multiple items to be chunked together, but also by strengthening the representations of individual items being held in memory. These findings provide new insight into the dynamic relationship between representations held in working and long-term memory.

## Supporting information

S1 Materials (DOCX)
