# Supplementary material for: Semantic knowledge influences visual working memory in adults and children
Source: PLoS One. 2020 Nov 11;15(11):e0241110. doi: 10.1371/journal.pone.0241110 (PMC7657485; doi:10.1371/journal.pone.0241110)
Supplement: S1 Materials — (DOCX) [file pone.0241110.s001.docx]

**Supplemental materials for Semantic Knowledge Influences Visual Working Memory in Adults and Children**

This supplement contains a summary of four additional experiments that were run in young adult participants prior to the Experiment 1 reported in the main manuscript. In Supplemental Experiment 1, participants performed a change detection task with pictures of either familiar or unfamiliar objects with a range of encoding durations. In Supplemental Experiment 2, we controlled for possible effects of verbal labeling strategies by having adult participants perform the same task, this time with a concurrent verbal dual task. In Supplemental Experiment 3, participants performed the change detection task with all of the images inverted to control for low-level visual features that may have differed between the familiar and unfamiliar objects. In Supplemental Experiment 4, participants were encouraged to verbally label the stimuli and encoding was self-paced. The results from these four supplemental experiments all support the conclusions of the primary manuscript.

**Supplemental Experiment 1**

Twenty participants completed Experiment 1 (mean age = 20.8 years). They saw an array of five pictures (all depicting either familiar or unfamiliar objects) that appeared on the screen for 300 ms, 1000 ms, or 2000 ms. After a 700 ms delay, one location was cued for 500 ms, after which the probe item appeared in that location. Participants completed six blocks of 33 trials each. Within each block, the stimulus type and encoding duration were held constant. Stimulus type and encoding duration were fully crossed, such that participants completed one block of each stimulus type at each encoding duration. Blocks were presented in a pseudorandom order, such that stimulus type alternated between blocks.

We analyzed accuracy data using a mixed effects model with stimulus type (familiar vs. unfamiliar object), encoding duration (300, 1000, or 2000 ms), and their interaction as possible fixed effects, and subject as a random effect. This analysis revealed significant effects of stimulus type, $\chi^{2}$ = 5.05, $p$ = 0.025, and encoding duration, $\chi^{2}$ = 81.08, $p$ < .001 (Supplemental Figure 1). The interaction was not significant, $\chi^{2}$ = 0.84, $p$ = 0.36. Memory performance was better for familiar objects, $M$ = 82.81%; 95% CI = [81.15 84.48], than unfamiliar objects, $M$ = 80.05% [78.29 81.81], and memory accuracy improved for both picture types as encoding duration increased. This result demonstrates that there is an advantage in working memory for remembering familiar compared to unfamiliar real-world objects.

Supplemental Figure 1. Visual working memory accuracy in adults for familiar versus unfamiliar objects. Error bars indicate SEM.

**Supplemental Experiment 2**

Supplemental Experiment 1 suggests that adults exhibit better visual working memory performance for familiar objects compared to unfamiliar objects. In Supplemental Experiment 2, we tested whether this familiarity advantage could have arisen from the use of verbal labeling strategies. If participants were attempting to label the objects, they would be more readily able to access labels for the familiar compared to unfamiliar objects, which would enable them to use verbal working memory in addition to visual working memory for the familiar arrays. To control for this possibility, we had participants perform a simultaneous verbal task of rehearsing two digits while performing the change detection task.

Twenty participants completed this experiment (mean age = 20.7 years). The procedure was the same as in Supplemental Experiment 1, with the exception that participants performed a concurrent verbal task intended to prevent them from verbally encoding the objects (Brady et al, 2016). Before each trial, participants were shown a two-digit number and were asked to covertly rehearse the digits throughout the trial. After the memory test, participants were prompted to type in the digits.

Participants exhibited good recall on the verbal digit task, $M$ = 86.24% [85.16 87.31]. For trials in which the digits were entered correctly, we analyzed memory accuracy data using the same type of mixed effects model as in Supplemental Experiment 1, with stimulus type, encoding duration, and their interaction as possible fixed effects, and subject as a random effect. This analysis again revealed significant effects of stimulus type, $\chi^{2}$ = 5.66, $p$ = 0.017, and encoding duration, $\chi^{2}$ = 4.93, $p$ = 0.026 (Supplemental Figure 2). The interaction was not significant, $\chi^{2}$ = 1.46, $p$ = 0.227. As in Supplemental Experiment 1, memory performance was better for familiar objects, $M$ = 85.93% [84.29 87.57], compared to unfamiliar objects, $M$ = 83.11% [81.31 84.9], and improved with increasing encoding duration. Thus, the verbal distractor task did not influence the effect of object familiarity on visual working memory performance, which suggests that the mnemonic benefit for familiar compared to unfamiliar objects in working memory does not stem from the use of a labeling strategy.

Supplemental Figure 2. Visual working memory accuracy in adults for familiar versus unfamiliar objects with a concurrent verbal dual task. Error bars indicate SEM.

**Supplemental Experiment 3**

In Supplemental Experiment 3, we investigated whether possible differences in low-level visual features between the familiar and unfamiliar objects that may have benefited memory performance for the familiar objects. To this end, we presented all of the images in an inverted orientation (see Curby et al., 2009). If the difference in memory performance found in Experiments 1 and 2 is due to low-level differences in the visual properties between the stimulus sets, then inverting the images should have no effect on the results. On the other hand, if the observed difference arises from the ability to access semantic knowledge about familiar objects, then inverting the images should slow down this process and attenuate the benefit for familiar objects (Curby et al., 2009).

Twenty-one participants completed this experiment (mean age = 21.9 years). The procedure and stimuli were the same as in Supplemental Experiment 1, with the exception that all of the stimuli were presented upside-down. We again analyzed memory accuracy data using a mixed effects model with stimulus type, encoding duration, and their interaction as possible fixed effects, and subject as a random effect. This analysis revealed a significant effect of encoding duration, $\chi^{2}$ = 110.29, $p$ = < .001 (Supplemental Figure 3). The main effect of stimulus type was not significant, $\chi^{2}$ = 3.06, $p$ = 0.08; nor was the interaction, $\chi^{2}$ = 0.05, $p$ = 0.831. In addition, a t-test comparing memory accuracy for familiar vs. unfamiliar items with the 2000 ms encoding window revealed no difference in memory performance, $t$(38) = 0.9, $p$ = 0.376, Cohen’s $d$ = 0.28. By contrast, the same t-test performed on the data from Supplemental Experiment 1, with the images in their original upright orientation, indicated that performance was significantly better for familiar objects compared to unfamiliar objects, $t$(38) = 2.14, $p$ = 0.039, Cohen’s $d$ = 0.68. These data militate against the idea that low-level differences in visual features between the familiar and unfamiliar stimuli drove the observed familiarity advantage in visual working memory in Supplemental Experiments 1 and 2.

Supplemental Figure 3. Visual working memory accuracy in adults for familiar versus unfamiliar objects with the images presented upside-down. Error bars indicate SEM.

**Supplemental Experiment 4**

In this experiment we further probed the effects of labeling strategies on visual working memory performance for familiar and unfamiliar objects. In this version, the encoding was self-paced: participants were allowed to view the stimulus arrays for up to one minute.

23 participants participated in this experiment (mean age: 20.6 years). On each trial, an array of 5 familiar or unfamiliar objects, or colored squares, appeared on the screen. The array remained on the screen until the participant pressed the space bar. After a 700ms delay, one location was cued for 500ms, after which the probe item appeared in that location. Participants pressed ‘S’ if the item was the same as the item that had previously been shown in the cued location, and ‘D’ if the item was different. Participants performed 100 trials, and the order of the different stimulus types (colored squares, familiar objects, and unfamiliar objects) was randomized.

The analysis of memory performance revealed a significant effect of stimulus type ($\chi^{2}$ = 49.00, $p$ < .001). Follow-up Tukey comparisons indicated that memory in both the familiar object (M = 95.9%; SE = 0.73) and unfamiliar object (M = 93.6%, SE = 0.89) conditions were better than memory in the color condition (M = 86.8%, SE = 1.24), but memory accuracy did not differ between the familiar and unfamiliar object conditions. The analysis of participants’ self-paced encoding time also revealed a significant effect of stimulus type ($\chi^{2}$ = 84.40, $p$ < .001). Participants chose to view the arrays of colored squares (M = 5.99 s, SE = .17) for less time than the arrays of familiar objects (M = 6.68 s, SE = .19), and chose to view the arrays of familiar objects for less time than the unfamiliar objects (M = 8.05 s, SE = .24).

These results demonstrate that although participants can use labeling to improve VWM memory and overcome the familiarity benefit, labeling strategies are time-intensive: participants chose to view the familiar items for more than 6 seconds, and the unfamiliar items for 8 seconds. Therefore, it is unlikely that participants were able to successfully engage in labeling strategies during the 500-2000 ms encoding durations used previous experiments, particularly given that participants were also performing a verbal digit repetition task in Supplemental Experiment 2.


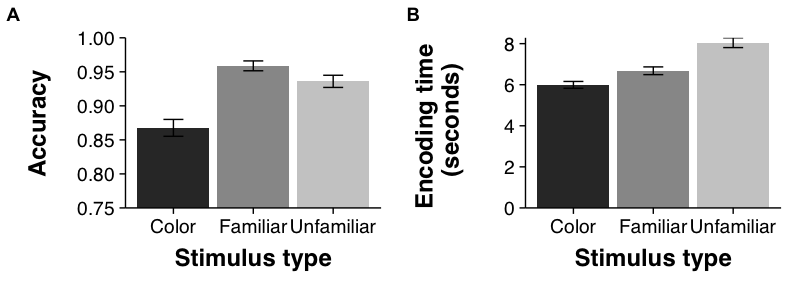


Supplemental Figure 4. Mean memory accuracy (A) and self-paced encoding time (B). Error bars indicate SEM.
